# Kyasanur forest disease virus: viremia and challenge studies in monkeys with evidence of cross-protection by Langat virus infection

**DOI:** 10.12688/f1000research.1-61.v1

**Published:** 2012-12-07

**Authors:** Keerti V Shah, Chandu N Dandawate, Pravin N Bhatt

**Affiliations:** 1 Department of Molecular Microbiology and Immunology, Johns Hopkins Bloomberg School of Public Health, Baltimore, MD, 21205, USA; 2 National Institute of Virology, Pune, MH 411001, India; 3 Section of Comparative Medicine, Yale University School of Medicine, New Haven, CT, 06520, USA

## Abstract

Kyasanur Forest Disease Virus (KFDV), discovered in 1957, is a member of the tick-borne encephalitis virus (TBEV) complex. Diseases caused by members of the TBEV complex occur in many parts of the world. KFDV produces a hemorrhagic fever in humans in South India and fatal illnesses in both species of monkeys in the area, the black faced langur (Presbytis entellus) and the bonnet macaque (Macaca radiata). Experimental infection of the langur and the bonnet macaque with early mouse passage KFDV strain P9605 resulted in a viremia of up to 11 days duration, peak viremia titers as high as 10 ^9^, and death in 82 = 100% of the animals. Prolonged passage of the KFDV strain P9605 in monkey kidney tissue culture resulted in a markedly reduced virulence of the virus for both species; peak viremia titers in monkeys decreased by 2.5 to 4.0 log LD 50 (p= 0.001), and the mortality decreased to 10% (p= 0.001). In challenge experiments, monkeys previously infected with tissue-culture-adapted KFDV, or with the related Langat virus from Malaysia, were fully protected against virulent KFDV. These studies in non-human primates lend support to the idea that a live virus vaccine from a member of the TBEV complex may be broadly protective against infections by other members of the TBEV complex.

## Introduction

Flaviviruses of the tick-borne encephalitis virus (TBEV) complex are widely and focally distributed in nature. New viruses of the complex and illnesses associated with them are frequently being described from different parts of the world. Human illnesses associated with infections with these viruses include mild or severe encephalitis and hemorrhagic fevers ^1^. Kyasanur Forest Disease Virus (KFDV), a member of the hemorrhagic fever group, was discovered in 1957 in the Shimoga district of Karnataka State, India, where it produced a febrile illness and deaths in humans, and deaths in both species of monkeys, the black-faced langur ( *Presbytis entellus*) and the red-faced bonnet macaque ( *Macaca radiata*), that inhabit the endemic area ^2^. Most of the human infections with KFDV occur in the drier months of the year, with peaks in March and April, and the number of cases is variable, but may reach over 500 in some years. Alkhurma Hemorrhagic Fever Virus (AHFV), described in 1997, a cause of severe hemorrhagic fever in Saudi Arabia and other regions of the Middle East ^3, 4^ and Nanjianyin Virus recovered from a febrile patient in Yunnan, China ^5^, are closely related to KFDV. Omsk Hemorrhagic Fever Virus (OHFV), which is endemic in western Siberia, Russia, and recognized as a tick-borne flavivirus in 1947, is distantly related to KFDV ^1^. In addition to the hemorrhagic fevers noted above, viruses of the TBEV complex are also responsible for over 10,000 cases of encephalitis, annually, in Europe and Asia, and for localized illnesses in different parts of the world ^1^.

In 1958–59, a formalin-inactivated mouse-brain virus vaccine prepared with the heterologous Russian spring-summer encephalitis virus (RSSE) was administered to over 10,000 residents in the KFDV-endemic area. Although this vaccine was protective against KFDV challenge in the mouse model ^6^, it was completely ineffective in preventing human KFDV infections in the endemic area ^7^. It also failed to prevent KFDV infections in laboratory personnel ^8, 9^. Serologically, individuals immunized with the RSSE vaccine showed a barely detectable immune response to KFDV antigens ^10^. In contrast to the lack of effectiveness of an inactivated RSSE vaccine, an inactivated KFDV vaccine reduced the incidence of KFDV in the affected area ^11^. Thus, KFDV infections in humans were preventable by an inactivated homologous vaccine, but not by an inactivated heterologous vaccine.

In the late 1950s and 1960s, investigators from the Virus Research Centre (VRC) [now the National Institute of Virology (NIV)] in Pune, India, conducted infection and challenge experiments in non-human primates using virulent and tissue-culture-adapted (TC-adapted) strains of KFDV as well as Langat virus (LGTV), a related virus of the TBEV complex isolated from ticks in Malaysia ^12^. The results of these studies were described only in the annual reports of the VRC and in a doctoral thesis published in 1963 ^6^, so they are not readily available. Because data obtained from experimental infection and challenge of non-human primates with viruses of the TBEV complex are valuable and may help efforts aimed at developing prophylactic vaccines, we have combined the information from the doctoral thesis and from an unpublished manuscript (Bhatt and Dandawate, unpublished observations, 1971) for this report.

We show here that 1) KFDV produces a high-titered viremia and death in both species of monkeys that inhabit the endemic area, 2) a TC-adapted KFDV has markedly reduced virulence for both monkey species, and 3) monkeys previously infected with TC-adapted KFDV or with heterologous LGTV completely resist challenge with virulent KFDV. Together, these data lend support to the idea that in contrast to the inactivated vaccines that do not cross-protect, an attenuated live virus vaccine derived from a member of the TBEV complex may be broadly protective against the different viruses of the complex.

## Materials and methods

### Monkeys

Studies described here were conducted at the VRC between 1958 and 1970.

The animals were trapped by professional catchers in the forested areas of Shimoga district, Karnataka State in south India, outside the known area of KFD infection, and were transported to the laboratory in Pune by train or by truck. The animals were held for observation for several weeks prior to inoculation. At the commencement of the experiments, the weight range (and mean weight) of the animals was 2.0–7.2 kg (4.6 kg) for langurs, and 1.8–5.6 kg (3.3 kg) for bonnets. Animals were first classed by weight and then individuals in each weight class were allocated randomly to the different groups in the experiment.

Pre-inoculation serum specimens from the animals were screened for antibodies to KFDV, LGTV, West Nile and dengue 2 by hemagglutination inhibition (HI) tests ^13^. Sera that had HI antibodies to any of the viruses (less than 10% of the sera) were screened for neutralizing antibodies to KFDV ^10^ and the rare animal that had KFDV neutralizing antibodies was not included in the experiment. The animals were inoculated with KFDV by the subcutaneous (SC) or the intravenous (IV) route and with LGTV by the IV or intracerebral (IC) route. The inoculated animals were monitored daily for viremia and general state of health. Rectal temperature was taken daily but these data were found to be uninformative and are not described in this report. Tissues of some of the animals that died during the observation period were tested for virus. Pathological studies of tissues were not routinely performed.

Data are reported from a total of 72 animals: 42 langurs and 30 bonnets. Three animals that did not develop either viremia or antibodies following inoculation are excluded from analyses.

### Viruses

The virus preparations used and their designations are listed below.

a) *Virulent KFDV.* Early passage KFDV is designated as virulent KFDV. Two strains of virulent KFDV were used. Most of the experiments were performed with strain P9605 derived from serum of a febrile individual in the endemic area and isolated and maintained by infant mouse brain passage in Swiss albino strain of mice. Passages 4–12 in infant mouse brain were employed with passage 9 used for a majority of the animals. These preparations are referred to as virulent KFDV (mouse brain) in this report. A second strain, KFDV 603260–2, was isolated and maintained in the bonnet macaque after inoculation from a mixture of two KFDV-positive tick pools. Monkey serum from the second passage of this strain was used as inoculum for one bonnet and four langurs. This strain is referred to as virulent KFDV (monkey serum).

b) *Tissue-culture-adapted KFDV.* The virulent KFDV P9605 was passaged in primary rhesus or bonnet monkey kidney tissue culture (MKTC) prepared from healthy wild-caught animals which were non-immune to KFD, and was shown to be attenuated for mice as judged by the decrease in mortality in these animals following intraperitoneal inoculation ^14^. The four preparations used in the primate studies were all derived from P9605, which had been adapted to MKTC following one infant mouse brain and two chick embryo tissue culture passages. The 137 ^th^ and 138 ^th^ monkey kidney tissue culture passage virus was used in the studies described in the doctoral thesis ^6^. This preparation is designated ‘TC-adapted KFDV MK 137–138’. Preparations employed in the experiments in the unpublished observations of Bhatt and Dandawate (1971) were i) 177 ^th^ monkey kidney tissue culture passage (designated TC-adapted KFDV MK 177); ii) virus plaque purified by three passages in monkey kidney cell monolayers after 144 passages in monkey kidney tissue culture and then passaged twice again in monkey kidney tissue culture (designated TC-adapted KFDV, plaque); and iii) the plaque-purified virus passaged 41 times in chick embryo tissue culture (designated TC-adapted KFDV, CE 41).

c) *Langat virus LGTV.* The TP 21 isolate of LGTV derived from a tick pool was used in its 8 ^th^ and 9 ^th^ infant mouse brain passage (LGTV, M8 or M9), or as the 14 ^th^ chick embryo tissue culture passage following nine infant mouse brain passages (LGTV, CE14).

### Viremia

Heparinized blood or serum specimens were collected daily beginning with day 1 or day 2 post-inoculation (pi) until day 10 or 11 pi. They were titrated on the day of the bleeding and, if end points were not reached, an aliquot stored at -70°C was re-titrated at a later date. Titrations for KFDV were performed in adult mice intracerebrally, except in one experiment where they were determined by inoculation of chick embryo tissue culture tubes (which have a sensitivity similar to that of adult mice). Titrations for Langat virus were performed in infant mice intracerebrally. Viremia values are reported as the positive logarithms of the LD50 titer per 0.03 ml (when titrated in adult mice or tissue culture) or 0.02 ml (when titrated in infant mice for Langat virus) of the inoculum. If the undiluted serum killed three of six mice, the estimate of the LD50 titer was 10 ^0.0^. If it killed less than one half of the mice the titer was reported as “trace virus (T)”.

### Statistics

**Figure 1.**
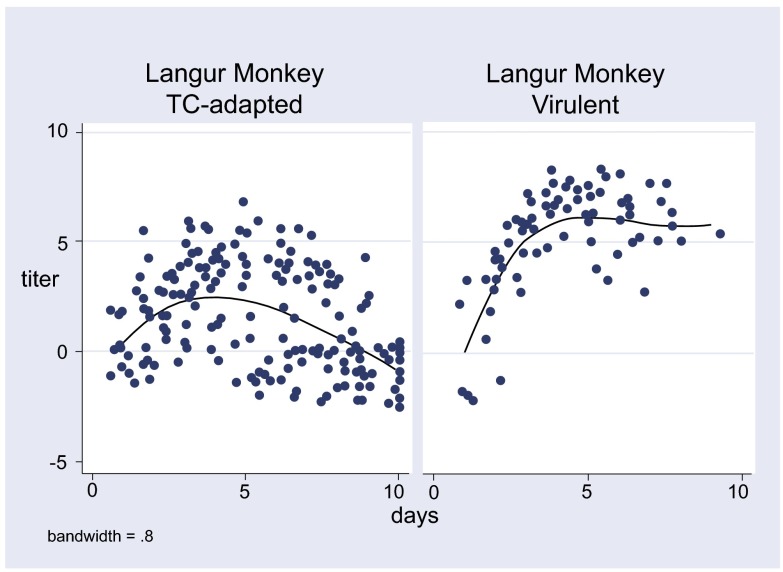
Viremia titers (log LD50) with TC-adapted and virulent KFDV in Langur monkeys.

**Figure 2.**
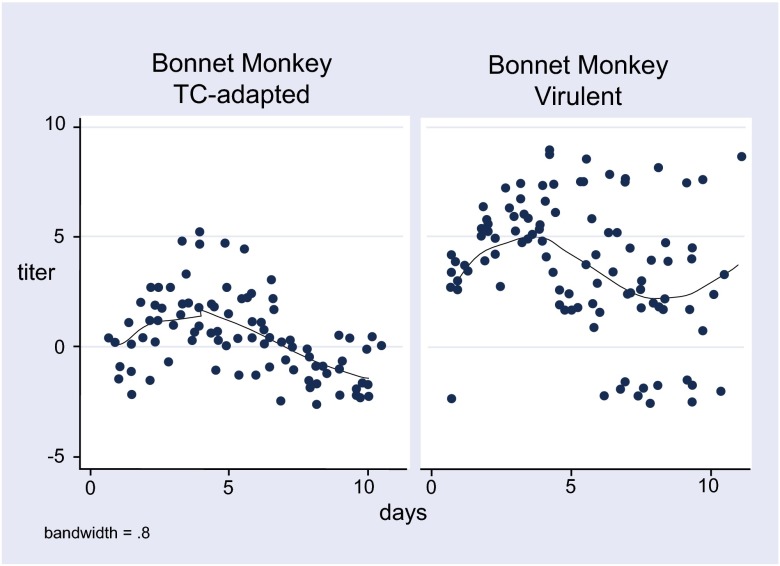
Viremia titers (log LD50) with TC-adapted and virulent KFDV in bonnet monkeys.

A lowess smoother was used to generate the estimated regression lines in Figure 1 and Figure 2. For continuous variables, non-parametric Wilcoxon test for differences between medians was used as a test of statistical significance. All graphs and statistical analyses were performed using Stata (version 9.0).

## Results

### Viremia and mortality in langur and bonnet monkeys with virulent and TC-adapted KFDV

*Virulent KFDV in the black-faced langur.* Thirteen langurs were inoculated with virulent KFDV by the SC or the IV route ( Table 1). The amount of virus in the inoculum ranged from 2.8–5.1 log LD50. Viremia was detected continuously from day 2 pi to the day of death or the day before death for 12 of the 13 animals. Peak titers in individual monkeys were reached between the 3 ^rd^ and 7 ^th^ day and they ranged from 10 ^4.5^–10 ^8.5^ with a median value (one value for each animal) of 10 ^6.6^.

**Table 1.** Viremia and mortality in the black-faced langur after infection with virulent KFDV. * Positive logarithms of adult mouse LD50 titers/0.03 ml.

Passage	Dose*/Route	Monkey Number	LD50 Titer* Day post-inoculation									
			1	2	3	4	5	6	7	8	9	10
Mouse brain	5.1/IV	166		4.6	5.5	6.6	X					
Mouse brain	5.1/IV	209		4.6	6.3	6.4	4.8	4.6	X			
Mouse brain	5.1/IV	210		3.5	6.1	6.5	5.9	X				
Mouse brain	5.1/IV	221		4.6	6.3	7.5	6.6	7.3	7.6	7.5X		
Mouse brain	5.1/IV	223		4.9	6.2	6.5	6.2	6.6	X			
Monkey serum	4.7/IV	84		2.4	4.5	4.4	3.7	3.0	2.2		X	
Monkey serum	4.7/IV	85		2.6	3.3	7.3	7.8	8.0X				
Monkey serum	2.8/SC	153	N	T	3.2	5.5	6.5	6.3	6.6	6.3X		
Monkey serum	2.8/SC	162	N	1.0	4.5	6.3	6.8	6.5X				
Monkey serum	4.1/SC	124	N	3.4	6.2		7.3	8.0X				
Mouse brain	4.7/SC	125	≥3.6	≥5.9	7.0	7.8	7.1	5.0	5.0	≥5.0	≥5.5	X
Mouse brain	4.2/SC	195	–	4.7	5.7	7.1	7.7	6.2	5.0	5.4	X	
Mouse brain	5.0/SC	291	2.6	4.2	6.7	7.9	8.5	X				

Peak titer is underlined.

T = Trace; N = Negative; X = Died; IV = Intravenous; SC = Subcutaneous.

All 13 animals died between day 5 and day 9 pi, with an average survival time of 6.5 days. With one exception, animals died during the viremic phase and the majority died at or near the peak of viremia. The exception was animal #84 that died on day 9, but for whom viremia was not estimated on the day of or day before death. Tissues of two animals collected post mortem (#84 and #85) were titrated for KFDV; all tested organs (blood, brain, lung, liver, spleen, kidney) were positive with titers ranging from 10 ^1.9^–10 ^6.5^.

*TC-adapted KFDV in the black-faced langur.* Twenty langurs were inoculated with TC-adapted KFDV by the IV or SC route. The amount of virus in the inoculum ranged from 10 ^1.9^ to 10 ^7.9^ ( Table 2). There was considerable variation in the duration and peak titers of viremia between animals given different passages of the TC-adapted KFDV. For example, four of the five animals given MKTC 138 had viremia of 5–6 days duration, and their peak titers did not exceed 10 ^3.6^. In contrast, viremia of 10 days duration, with peak titers of 10 ^5.0^ or greater, were seen in animals that received CE 41. The highest titers in the 20 individual animals (one value for each animal) ranged from 10 ^0.6^ to 10 ^5.7^ with a median value of 10 ^4.5^.

**Table 2.** Viremia and mortality in the black-faced langur after infection with TC-adapted KFDV. * Positive logarithms of adult mouse LD50 titers/0.03 ml.

Passage	Dose*/Route	Monkey Number	LD50 Titer* Day post-inoculation									
			1	2	3	4	5	6	7	8	9	10
MK138	5.9/IV	147		1.5	2.5	3.6	2.0	2.4	1.6	1.0	N	N
MK138	5.9/IV	171		1.7	2.6	1.6	1.2	0.7	0.2	N	T	N
MK138	5.9/IV	207		1.1	0.4	T	T	T	N	N	N	N
MK138	5.9/IV	220		0.5	0.4	0.6	T	0	N	T	N	N
MK138	5.9/IV	222		1.6	0.8	0.9	N	T	N	N	N	N
MK138	5.9/IV	225		0.5	3.1	3.2	4.3	X				
MK177	3.9/SC	22	N	2.0	3.1	4.2	4.4	3.5	3.2	2.7	T	T
MK177	2.9/SC	23	N	T	3.0	3.8	4.0	3.3	3.5	<2.0	T	N
MK177	1.9/C	33	N	T	<2.0	4.0	5.0	5.7	4.7	<3.0	<2.0	T
MK177	8.3/SC	30	T	3.3	4.5	3.3	T	T	N	N	N	N
MK177	8.3/SC	85	≥2.0	≥4.0	5.5	6.0	6.3	5.1	4.1	4.0	≥2.0	TX
MK177	4.0/SC	101	T	T	≥3.5	4.3	5.1	3.7	3.5	3.2	≥2.0	T
Plaque	7.9/SC	87	1.9	≥3.5	4.5	3.3	T	T	N	N	N	N
Plaque	7.9/SC	92	1.9	≥3.1	4.5	4.0	4.1	3.0	N	N	N	N
Plaque	3.9/SC	100	T	≥2.0	≥3.3	5.0	4.0	3.6	2.5	N	N	N
Plaque	3.9/SC	102	N	≥2.0	3.5	3.7	4.3	4.1	3.2	3.0	T	N
CE41	5.7/SC	193	T	≥3.5	4.0	4.8	5.0	4.0	3.8	T	4.0	T
CE41	5.7/SC	194	2.7	≥5.5	5.5	5.7	5.7	4.5	5.7	≥2.0	N	T
CE41	5.7/SC	197	T	2.3	3.7	3.4	3.5	T	T	N	N	N
CE41	5.7/SC	198	T	T	3.7	5.3	5.0	4.5	5.0	T	T	N

Peak titer is underlined.

T = Trace; N = Negative; X = Died; IV = Intravenous; SC = Subcutaneous.

There were two deaths among the 20 animals, one (#225) on day 6 at the peak of viremia and the other on day 10 (#85) in a monkey which was viremic from days 1–10. In tests of brain, lung, spleen, liver, kidney and heart tissues of animal #85, KFDV was isolated only from the lung tissue. The lung isolate was identified as the TC-adapted strain on the basis of its low pathogenicity for subadult mice ^14^.

*Virulent KFDV in the bonnet macaque.* Eleven bonnet macaques were inoculated with virulent KFDV by the IV or SC route. The amount of virus in the inoculum ranged from 10 ^4.0^ to 10 ^8.1^ LD50 ( Table 3). The duration of viremia after inoculation of the mouse brain virus was from 5 to 10+ days. Peak titers of between 10 ^4.2^ and 10 ^9.0^ were reached on the second, third or fourth day. In the single animal given the monkey serum virus, viremia was observed from days 2 to 11 with high titers ranging from 10 ^7.2^ to 10 ^9.0^ from days 5 to 11. The median value of the highest titers in the 11 bonnets was 10 ^6.5^.

**Table 3.** Infection and mortality in the bonnet macaque with virulent KFDV. * Positive logarithms of adult mouse LD50 titers/0.03 ml.

Passage	Dose*/Route	Monkey Number	LD50 Titers* Day post-inoculation										
			1	2	3	4	5	6	7	8	9	10	11
Mouse brain	7.6/IV	276	3.8	5.3	≥ 6.5	5.3	3.6	3.7	2.4	4.1	X		
Mouse brain	7.6/IV	277	3.4	4.0	5.3	3.8	2.1	1.5	T	N	N	N	
Mouse brain	7.6/IV	279	3.0	4.2	4.8	4.3	1.8	3.6	1.9	1.2	X		
Mouse brain	7.6/IV	281**	3.6	5.6	5.8	4.8	2.6	2.6	2.0	2.2	1.5	0.3	
Mouse brain	8.1/IV	302**	3.0	5.2	6.0	4.8	1.8	N	N	N	N		
Mouse brain	8.1/IV	311	4.0	5.2	6.0	5.8	≥2.0	1.5	N	N	N	X	
Mouse brain	8.1/IV	316	2.5	5.5	6.5	7.0	≥2.0	≥2.0	≥3.0	≥2.0	X		
Mouse brain	8.1/IV	325	4.2	≥6.7	≥ 7.2	7.0	≥2.0	≥2.0	≥3.0	≥2.0	X		
Monkey serum	4.0/SC	174	N	3.1	4.5	5.3	7.2	7.5	7.3	8.2	7.8	7.9	8.3X
Mouse brain	4.2/SC	123		4.8	6.4	7.5	7.5	5.0	3.9	4.1	4.0	2.1	
Mouse brain	4.2/SC	297		5.2	7.0	9.0	8.4	6.0	5.5	4.7	4.1	3.6	

Peak titer is underlined.

T = Trace; N = Negative; X = Died; **#281 died on day 21, #302 on day 20; IV = Intravenous; SC = Subcutaneous.

Nine of the 11 animals inoculated with the virulent virus died. Deaths occurred either at the end of the viremic phase (4 deaths on day 9 and one on day 10), or at the height of viremia (one death on day 11 with the monkey serum virus), or in the post-viremic phase (one death each on day 14, 20, and 21). Tissues of five of these animals were tested for KFDV. Three of these animals (#276, #279, #174) died early in the illness during the viremic phase; virus was recovered from all of their tissues (blood, liver, lung, spleen, kidney) that were examined. The amount of virus was highest in spleen or blood and lowest in the brain. In the two animals that died in the post-viremic phase at the end of the third week (#281 and #302), virus was present almost exclusively in the brain with titers of 10 ^4.2^ and 10 ^4.9^.

*TC-adapted KFDV in the bonnet macaque.* Ten animals were inoculated either by the IV or the SC route with 10 ^5.3^–10 ^6.3^ LD50 of the virus ( Table 4). The duration of viremia varied from 6–8 days. The peak titers in individual monkeys ranged from 10 ^1.6^–10 ^5.5^ and were obtained between 2 and 5 days pi. The median value of the highest titers in the 10 bonnets was 10 ^2.3^.

**Table 4.** Infection and mortality in the bonnet macaque with TC-adapted KFDV. * Positive logarithms of adult mouse LD50 titers/0.03 ml.

Passage	Dose*/Route	Monkey Number	LD50 Titer* Day post-inoculation									
			1	2	3	4	5	6	7	8	9	10
MK137	5.3/IV	256	0	0.6	2.2	1.5	0	0.6	N	N	N	N
MK137	5.3/IV	263	0	0.6	2.0	1.6	0.6	0.9	0	N	N	N
MK137	5.3/IV	285	N	1.0	1.7	2.3	2.3	1.6	0.2	T	T	N
MK138	6.3/IV	291	0.1	1.6	1.5	0.6	T	T	T	T		N
MK138	6.3/IV	294	1.4	≥ 2.4	1.9	1.0	0.5	0.1	N	N		N
MK138	6.3/IV	298	N	1.6	2.0	2.3	1.0	1.5	1.5	T		N
MK138	6.3/IV	307	0.3	1.6	1.1	0.7	0.5	0.5	0	N		N
CE41	5.7/IV	120	T	T	T	T	2.2	2.5	3.0	T	N	N
CE41	5.7/SC	121	T	2.5	3.5	5.5	4.5	2.5	2.0	N	N	N
CE41	5.7/SC	139**	T	3.0	4.5	5.0	4.5	T	T	N	N	N

Peak titer is underlined.

T = Trace; N = Negative; ** #139 died on day 26; IV = Intravenous; SC = Subcutaneous.

There was one death during the observation period. On day 26, animal #139 which was viremic for 7 days pi died after a bout of diarrhea. In tests of the tissues of this animal for virus, lung, liver, heart and kidney were negative but less than 10 LD50 of virus were recovered from brain tissue. The isolate was identified as attenuated by its low pathogenicity in adult mice. The isolate when inoculated in four bonnets each by the IC and SC routes produced viremia in all, but no death or overt encephalitis in any animal.

The viremia data of the virulent and TC-adapted strains of KFDV in the two species of monkeys are summarized in Figure 1 and Figure 2. As compared to the virulent strain, the TC-adapted strain had 2.15 log10 units lower peak titers in the langur and 4.0 log10 units lower peak titers in the bonnet macaque. These differences were highly significant (p < 0.001) ( Table 5). The mortality in the two species was reduced from 82–100% for the virulent strain to 10% for the TC-adapted strain (p < 0.001) ( Table 5).

**Table 5.** Summary of viremia and mortality data of virulent and TC-adapted KFDV in langur and bonnet monkeys. * One value for each animal, underlined in Table 1– Table 4.

Species	KFDV	Highest viremia titer, log LD50			
		Median (range)	P value	Mortality	P value
Langur	Virulent	6.6 (4.5–8.5)		13/13	
	TC-adapted	4.5 (0.6–6.3)	p<0.001	2/20	p<0.001
Bonnet	Virulent	6.5 (4.8–9.0)		9/11	
	TC-adapted	2.3(1.6–5.5)	p=0.001	1/11	p=0.001

### Infection of langur and bonnet monkeys with Langat Virus

*In the black-faced langur.* Peak titers in individual monkeys inoculated with the ninth infant mouse brain passage of the TP21 strain of Langat virus ranged from 10 ^0.5^–10 ^1.5^ and were observed on days 2 or 3 pi. The duration of viremia varied from 2–7 days with a mean duration of 4.2 days. Four animals inoculated IC with the tissue culture virus had a somewhat longer duration of viremia (mean duration 5.8 days), peak titers were observed on days 3 and 4 pi and they ranged from 10 ^1.6^–10 ^2^.

*In the bonnet macaque.* All nine animals inoculated either IV (5 animals) or IC (4 animals) with Langat TP21 M8-M9 were viremic on the first two days; peak titers ranging from 10 ^1.6^ to >10 ^4.0^ (mean of 10 ^2.6^) were observed. The proportion of monkeys circulating the virus as well as the level of viremia decreased after the second day and viremia was not demonstrable in any monkey after day 6. The average duration of viremia was 3.6 days.

### Challenge of previously infected animals with virulent KFDV

Groups of animals, previously infected with Langat virus or with TC-adapted KFDV, were challenged with virulent KFDV. Data are combined in Table 6.

**Table 6.** Challenge of immunized monkeys with virulent KFDV.

Immunization			Challenge		Viremia and mortality	
Virus	Species	N	Time post-infection	Virulent KFDV Dose, log LD50/Route		
Langat	Bonnet ^1^	8	38 days	7.6/IV	Trace virus*	0/7
KFDV-TC adapted	Bonnet ^2^	4	31 days	8.1/IV	None	0/4
KFDV-TC adapted	Langur ^3^	9	4–30 months	4.2–5.0/SC	None	0/9
KFDV-TC adapted	Bonnet ^4^	2	4–30 months	4.2/SC	None	0/2
None	Bonnet ^5^	8			All viremic 2–10 days	7/8
None	Langur ^6^	3			All viremic 5–9 days	3/3

^1^Survivors of IV or IC infection.

^2^Survivors of animals infected with MK137 and MK138 in Table 4.

^3^Survivors of animals infected with MK177, plaque and CE41 in Table 2.

^4^Survivors of animals infected with CE41 in Table 4.

^5^Described in Table 3, top 8 animals.

^6^Described in Table 1, last 3 animals.

IV = Intravenous; SC = Subcutaneous.

Eight bonnets previously infected with Langat virus, and 4 bonnets previously infected with TC-adapted KFDV were challenged IV with 7.6–8.1 log LD50 of virulent KFDV. The interval between the immunizing infection and challenge was 31–38 days. In daily tests for viremia for 10 days, no viremia was detected in the four animals previously infected with TC-adapted KFDV, but ‘trace’ viremia was detected in 3 of the 8 bonnets previously infected with Langat virus, on day 1 or 2 pi. All but one animal survived challenge. One bonnet previously infected with Langat virus died on day 10 after challenge; blood, liver, lung, kidney, spleen and brain of this animal did not yield virus. This death was considered unrelated to the challenge.

In a second series of challenge experiments in the unpublished observations of Bhatt and Dandawate (1971), 9 langurs and 2 bonnets previously infected with TC-adapted KFDV were challenged with 4.2–5.0 log LD50 of virulent KFDV. The interval between the immunizing and challenge infections was 4–30 months. In daily tests for viremia for 10 days pi, none were shown to be viremic. All of the animals survived the challenge.

## Discussion

Kenyon *et al.* ^15^ have stated that “one of the most serious constraints for the study of TBE pathogenesis and for vaccine development has been the absence of realistic animal models that mimic human disease”. Our results of infection of two species of monkeys with virulent and TC-adapted KFDV and with Langat virus, and challenge of survivors with virulent KFDV, address this question.

## Animal model for KFDV pathogenesis

Both the black-faced langur and the red-faced bonnet appear to be good models to study KFDV infection. Both species are naturally infected by KFDV in the endemic forested area in south India and many succumb to the disease ^16, 17^. A large majority of the animals found dead in the forest are langurs. The results of our experimental infections showed that the langur was highly susceptible to KFDV, and that the disease was acute, of short duration and always fatal. Viremia titers were high and deaths occurred between days 5–10 pi at or near the peak of viremia. Disease in the bonnet macaque seemed to resemble more closely the biphasic human disease. In some infected humans, the acute viremic phase is followed by a second non-viremic phase in which the patient has neurological symptoms and abnormal cerebrospinal fluid, but the symptoms are not localizing and there are no neurologic sequelae ^18, 19^. In the bonnet macaque, some deaths occurred during the viremic phase, but others occurred in the third week, at which time virus was recovered from the brain but not from other organs. In previous investigations, Webb and Burston ^20^ have described neurological changes in the post-viremic period in experimentally infected bonnet macaques. Kenyon *et al.* ^15^ have reported that the bonnet macaques experimentally infected with KFDV have a 100% mortality and that they show significant hematological and neurological abnormalities similar to those in KFDV-infected humans as well as significant histopathological changes in the small and large intestine, spleen and lymph nodes.

In contrast to its high pathogenicity in the bonnet macaque, KFDV does not produce illness or death in the rhesus macaque, although the pattern of viremia is similar in the two species ^2, 6, 21^.

*Reduced virulence of TC-adapted KFDV*: Bhatt and Anderson ^14^ have reported that the passage of the prototype P9605 strain of KFDV in monkey kidney tissue culture (MKTC) resulted in a decrease of the virulence of the virus for adult mice as judged by a decrease in LD50 titer of the virus following intraperitoneal (IP) inoculation. In the current investigation, the lower virulence of the virus was also clearly evident in both species of monkeys. In the langurs, the viremia levels seemed to be lower after infection with MKTC 138 passage virus as compared to titers after inoculation of the other three preparations of TC-adapted KFDV. Nevertheless, combined for all passage levels and for both species, the mortality decreased from 82–100% with the virulent virus to 10% with the TC-adapted virus. The reduced virulence was also reflected in a decrease in peak titers of viremia of 2.5–4.5 log LD50 in the two species of monkeys. Identification of changes in the KFDV genome associated with this decrease in virulence may help us to understand the genetic determinants of KFDV virulence.

## Protection against homologous and heterologous challenge

Viruses of the TBE complex are distributed focally in many parts of the world and they produce diseases of varying severity and in varying numbers. So, a single vaccine which can provide protection against all members of the complex would be beneficial. Among viruses of the TBE complex, Langat virus from Malaysia is the least virulent for mice, and mice infected intraperitoneally with Langat virus survive and they resist IP challenge by all of the viruses of the TBE complex that were tested ^22^. Monkeys that had survived infection with the TC-adapted KFDV or with Langat virus completely resisted challenge with virulent KFDV. There was not a single virus-related death in these animals. In addition, there was no viremia at all in the animals previously infected with attenuated KFDV and only traces of viremia in some of the monkeys previously infected with Langat virus. A similar protection against homologous as well as heterologous challenge has been demonstrated in a report by Rumyantsev *et al.* ^23^ in which chimeric live virus vaccines which contained sequences of TBEV and dengue 4 (TBEV/den4) or Langat virus and dengue 4 (LGT/den 4) were tested in rhesus monkeys. Animals immunized with either of the chimeric vaccine did not develop viremia when challenged with Langat virus.

Live Langat virus has been used in experimental treatment of human malignant disease in the United Kingdom and also as a live virus vaccine against TBE in Russia. In both cases, the neurotropism of Langat virus for humans became apparent. In the United Kingdom, two of 23 cancer patients infected with Langat virus developed encephalitis 8–11 days after the end of viremia ^24^. Immunization of over 600,000 individuals with live Langat virus was reported to have reduced markedly the incidence of encephalitis in the endemic region in Russia, but the vaccine was associated with a serious adverse effect and was responsible for the occurrence of 35 cases of meningoencephalitis with permanent sequelae ^1^. Attempts have been made to construct Langat virus which retains its immunogenicity but is less neuroinvasive ^25^.

The TC-adapted strain of KFDV has a markedly decreased virulence for non-human primates and it merits consideration for further development as a live virus vaccine. It may be anticipated that an attenuated KFDV vaccine will provide significantly greater protection against KFD than inactivated KFDV vaccine, and it may also be effective against other viruses of the TBE complex. An attenuated KFDV vaccine may be less likely to produce paralytic sequelae than the Langat live virus vaccine because human KFDV infections in south India, while they cause transient neurological illness in some infected individuals, are not associated with paralytic disease. Components of the attenuated KFDV strain could also be employed to construct a genetically engineered non-infectious recombinant TBEV vaccine ^23, 26^ in the search for a safe and broadly effective vaccine strategy against viruses of the TBEV complex.
