# Supplementary material for: Kyasanur forest disease virus: viremia and challenge studies in monkeys with evidence of cross-protection by Langat virus infection
Source: F1000Res. 2012 Dec 7;1:61. [Version 1] doi: 10.12688/f1000research.1-61.v1 (PMC3924949; doi:10.12688/f1000research.1-61.v1)
Supplement: Peer review history

# Peer review: Referee response for version 1

**Published:** 2012-12-19

**Authors:** Alexander Freiberg

**Affiliations:** 1 Department of Pathology, University of Texas Medical Branch, Galveston, TX, USA

I have read this submission. I believe that I have an appropriate level of expertise to confirm that it is of an acceptable scientific standard.

# Peer review: Referee response for version 1

**Published:** 2012-12-18

**Authors:** Michael Holbrook

**Affiliations:** 1 National Institute of Health (NIH), Bethesda, MD, USA

I have read this submission. I believe that I have an appropriate level of expertise to confirm that it is of an acceptable scientific standard.

# Peer review: Referee response for version 1

**Published:** 2012-12-17

**Authors:** Dennis A Bente

**Affiliations:** 1 Departments of Microbiology and Immunology and Internal Medicine, The University of Texas Medical Branch, Galveston, TX, USA

I have read this submission. I believe that I have an appropriate level of expertise to confirm that it is of an acceptable scientific standard.
